# Computed Tomography of the Head: A Systematic Review on Acquisition and Reconstruction Techniques to Reduce Radiation Dose

**DOI:** 10.1007/s00062-023-01271-5

**Published:** 2023-03-02

**Authors:** Michael Dieckmeyer, Nico Sollmann, Karina Kupfer, Maximilian T. Löffler, Karolin J. Paprottka, Jan S. Kirschke, Thomas Baum

**Affiliations:** 1 Department of Diagnostic, Interventional and Pediatric Radiology, Inselspital, Bern University Hospital, University of Bern, Bern, Switzerland; 2 Department of Diagnostic and Interventional Neuroradiology, School of Medicine, Klinikum rechts der Isar, Technical University of Munich, Munich, Germany; 3 TUM-Neuroimaging Center, Klinikum rechts der Isar, Technical University of Munich, Munich, Germany; 4 Department of Diagnostic and Interventional Radiology, University Hospital Ulm, Ulm, Germany; 5 Department of Diagnostic and Interventional Radiology, University Medical Center Freiburg, Freiburg im Breisgau, Germany

**Keywords:** Iterative reconstruction, Dose reduction, Low dose, Radiation exposure, Head CT, CT angiography

## Abstract

In 1971, the first computed tomography (CT) scan was performed on a patient’s brain. Clinical CT systems were introduced in 1974 and dedicated to head imaging only. New technological developments, broader availability, and the clinical success of CT led to a steady growth in examination numbers. Most frequent indications for non-contrast CT (NCCT) of the head include the assessment of ischemia and stroke, intracranial hemorrhage and trauma, while CT angiography (CTA) has become the standard for first-line cerebrovascular evaluation; however, resulting improvements in patient management and clinical outcomes come at the cost of radiation exposure, increasing the risk for secondary morbidity. Therefore, radiation dose optimization should always be part of technical advancements in CT imaging but how can the dose be optimized? What dose reduction can be achieved without compromising diagnostic value, and what is the potential of the upcoming technologies artificial intelligence and photon counting CT? In this article, we look for answers to these questions by reviewing dose reduction techniques with respect to the major clinical indications of NCCT and CTA of the head, including a brief perspective on what to expect from current and future developments in CT technology with respect to radiation dose optimization.

## Key Points

1. Advanced iterative reconstruction enables significant dose reduction in head CT.
2. For CTA of the head, the combination with lower CM volume can be particularly effective for improved patient care.
3. AI and PCCT can be expected to enable additional dose reductions of at least 40%.
4. For novel techniques, careful evaluation of image quality and diagnostic performance is essential to ensure diagnostic benefits head CT.

## Introduction

Over the last decades, we have seen a steady growth in the number of computed tomography (CT) examinations [1–3]. New technological developments, broader availability of the required hardware and software, as well as physician and patient demands are the main reasons for increased clinical application of CT [1, 2]. Although magnetic resonance imaging (MRI) has developed tremendously over the last decades, in particular for head imaging, and has become more available, CT still is the workhorse of head imaging for multiple indications, including non-contrast CT (NCCT) in cerebral ischemia and stroke, assessment of intracranial hemorrhage (ICH), headaches, or acute neurologic deficits, as well as for first-line diagnostics in loss of consciousness and trauma evaluation in the emergency setting [4]. Detailed assessment of cerebral blood supply has become the clinical standard through the use of CT angiography (CTA) and CT perfusion (CTP) [5, 6]. Therefore, CT of the head is essential for fast and accurate diagnosis, optimized patient management and treatment. Besides MRI, which is generally used in less acute clinical settings, CT makes up a large proportion of the daily neuroradiological workload in head imaging.

However, CT comes with the inherent downside of ionizing radiation, which may cause radiation-induced malignancies [7, 8]. In the USA, approximately 2% of future cancer cases are assumed to be attributable to the current application of medical imaging [3, 9], and CT exposure was estimated to be responsible for 1% of total cancer mortality [10]. Therefore, the principle to keep radiation exposure as low as reasonably achievable (ALARA) is fundamental [11, 12].

Unfortunately, CT-related radiation exposure in daily clinical routine still shows strong inter-institutional and intra-institutional variations, as well-defined and universal reference standards are usually not available [13, 14]. General recommendations are complicated to determine considering different scanner models and technologies, which considerably influence radiation exposure. Using the technique only when the clinical value outweighs risks and costs, and restricting the scan volume to the clinical task are undoubtedly the most effective ways to limit CT-related radiation exposure in order to protect patients. Besides a careful risk-benefit assessment, developments on both the acquisition and the reconstruction side have led to an optimized trade-off between image quality (IQ) and radiation exposure [15, 16]. A widely applied dose reduction approach is the optimization of acquisition parameters, including tube voltage, tube current, and contrast medium (CM) volume. Tube current is expressed either directly (as mA) or indirectly in terms of tube current-time product (as mAs). Modern clinical CT systems use automatic exposure control (AEC) by means of automatic tube current modulation (ATCM), which is based on patient habitus, z‑axis modulation, and rotational modulation [17–19].

Increased image noise and artifacts are the drawbacks of most dose reduction methods but could at least partially be compensated by adequate image reconstruction techniques. Developments in iterative reconstruction (IR) techniques have led to major improvements in recent years, which have therefore become an essential component of CT dose reduction [16]. Before the emergence of IR, filtered back projection (FBP) was the standard image reconstruction for clinical CT. This fast and robust method is based upon the exact mathematical relation between measured projection and reconstructed image data [16, 20]. However, particularly for dose-reduced acquisitions, FBP-reconstructed images can suffer from low quality, as noise-free data are assumed and image noise is amplified in the filtering process. In contrast, IR can reduce image noise through iterative filtering or modeling of data acquisition physics [16]. Currently, hybrid IR (HIR), such as iDose (Philips Healthcare, Best, The Netherlands), ASIR (GE Healthcare, Milwaukee, WI, USA), or SAFIRE (Siemens Healthineers, Erlangen, Germany), is the most frequently used IR technique, providing increased reduction of image noise and reconstruction time through iterative filtering of both projection and image data. Benefits in terms of achievable IQ, in particular at low doses, are only exceeded by model-based IR (MBIR) algorithms, which use advanced models in an iterative process of backward and forward projections. However, the exceptional level of noise reduction and achievable dose reduction comes at the cost of high computational efforts, limiting accessibility and usability in daily clinical practice. Yet, artificial intelligence (AI) could help to overcome this limitation by using a convolutional neural network (CNN) trained with (simulated) low-dose (LD) data to reconstruct standard-dose (SD) high-quality CT images [21–24].

Although the use of dose reduction techniques for head CT is increasing, they have not yet been systematically reviewed. Therefore, the purpose of this article was to review clinical applications of dose reduction and LD techniques for NCCT and CTA of the head. The objective was to analyze achievable dose reductions, effects on IQ, and diagnostic performance with respect to the most relevant pathologies that are frequently assessed by head CT applications. Availability and quality of the included literature has a high degree of heterogeneity with respect to dose reduction methods, reported dose parameters, and clinical applications. Therefore, we concentrated on five major clinical indications: (i) NCCT of cerebral ischemia and stroke, (ii) NCCT of intracranial hemorrhage, (iii) NCCT of the head without specified indications, (iv) CTA of intracranial aneurysms, and (v) CTA of other cerebrovascular diseases and carotid artery disease.

## Material and Methods

### Search Strategy

A search of PubMed (http://www.ncbi.nlm.nih.gov/pubmed) was performed to identify studies evaluating methods to reduce radiation dose for NCCT and CTA of the head with respect to the following clinical indications: (i) NCCT of cerebral ischemia and stroke, (ii) NCCT of ICH, (iii) CTA of intracranial aneurysms, and (iv) CTA of other cerebrovascular diseases and carotid artery disease. Additionally, NCCT studies without specified indications were identified by screening of references of included studies. The search was conducted by two persons (radiologists with 7 and 4 years of experience, respectively) without a beginning search date (search end date 19 July 2022). Uncertainties about inclusion of a respective article, if present, were resolved by consensus through discussion with a third person (board-certified consultant in radiology, 11 years of experience).

**Fig. 1:**
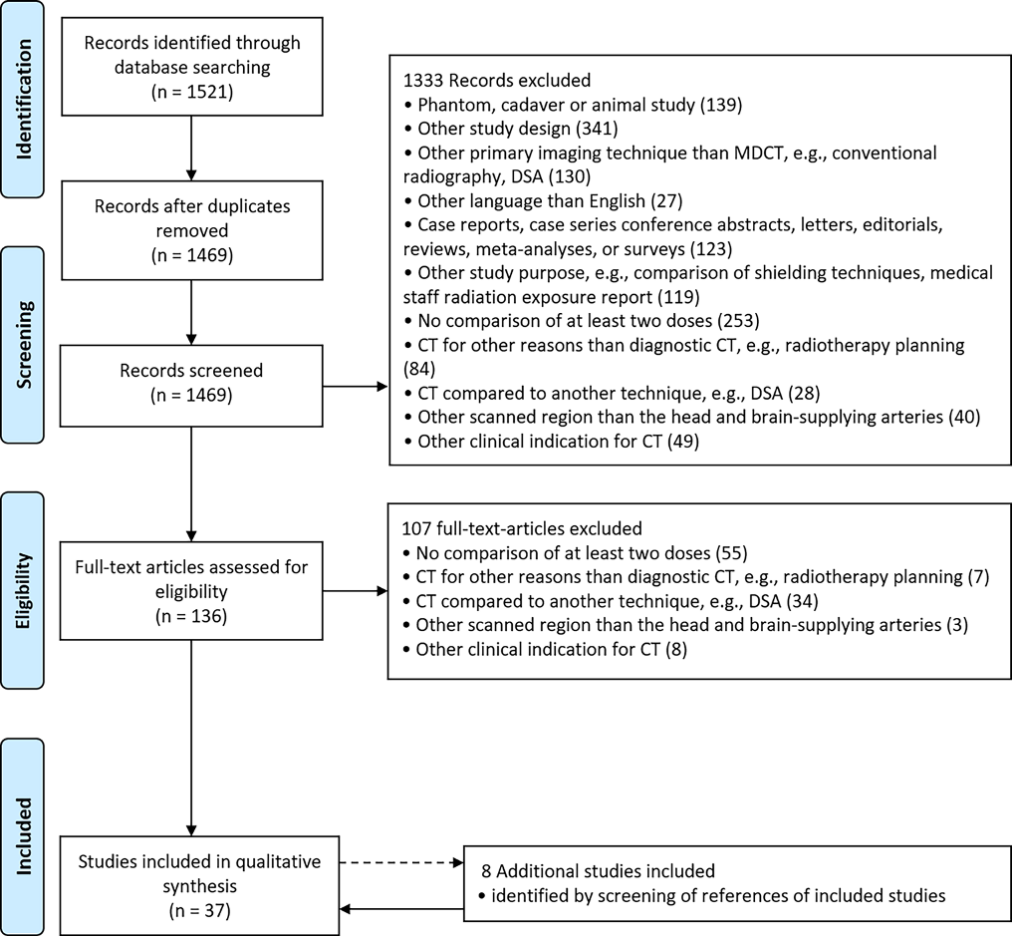
PubMed search flow diagram according to the Preferred Reporting Items for Systematic reviews and Meta-Analyses (PRISMA) guidelines [25, 26]

The literature search was performed according to the Preferred Reporting Items for Systematic reviews and Meta-Analyses (PRISMA) guidelines (Fig. 1; [25, 26]). The used search terms for PubMed are available in the appendix.

### Inclusion Criteria

Studies were included if they met the following inclusion criteria: (1) study population: studies performed in humans including adult or pediatric patients; (2) study design: retrospective or prospective; (3) indications and scanning type: diagnostic NCCT for present or suspected intracranial pathology, or CTA for evaluation of the intracranial vasculature and/or carotid arteries and (4) purpose: comparison of LD to SD protocols using CT data acquired at different dose levels, CT data acquired at a single dose level and additionally simulated at different dose levels, or CT data including a dose comparison between patient subgroups.

### Exclusion Criteria

Studies were not considered if they met the following exclusion criteria: (1) article type: case reports, case series, conference abstracts, letters, editorials, reviews, meta-analyses, or surveys; (2) language of publication other than English; (3) studies in cadavers, phantoms, or animals and (4) studies with other purposes (e.g., exclusive comparison of CM volumes, comparison of shielding techniques, medical staff radiation exposure reports).

### Extraction of Data

The following information was extracted from the selected articles: (1) author(s); (2) year of publication; (3) number of subjects (*n*) of the entire study and relevant patient subgroups (e.g., SD group, LD group); (4) details on group comparisons (if applicable); (5) details on the used CT system, including number of detector rows, vendor, and model name; (6) image acquisition parameters; (7) image reconstruction algorithms and parameters; (8) dose reduction (in %) and (9) reported dose values: CT dose index (CTDI_vol_), dose length product (DLP), and/or effective dose (E).

## Results

### Study Selection

The search via PubMed resulted in 1469 publications after removal of duplicates. During screening of titles and abstracts, 1333 records were discarded. The assessment of full-text articles led to the removal of 107 records, and the reference screening of included studies led to the addition of 8 articles, resulting in 37 publications that were included in the qualitative synthesis for this systematic review (Fig. 1).

### Study Characteristics

The 37 selected studies covered NCCT of cerebral ischemia and stroke (*n* = 6), NCCT of ICH (*n* = 4), NCCT without specified indications (*n* = 8), CTA of intracranial aneurysms (*n* = 10), and CTA of other cerebrovascular diseases (*n* = 10).

#### Patients

The total number of subjects (*n*) as well as the number of subjects in the SD group(s) and LD group(s) were extracted. Furthermore, the number of included CT examinations or subject numbers for relevant subgroups (e.g., used CT system, administered CM dose) were extracted when provided. Total numbers ranged from *n* = 20 [27] to *n* = 305 patients [28].

#### CT System, Acquisition and Reconstruction Parameters

All except one of the studies used multi-detector CT (MDCT). One study published in 2005 used single-detector CT [29]. Tube voltages from 70 to 150 kV were used. Dose reduction in LD protocols was achieved by a reduction of tube voltage, tube current, or by a combination of both. Reduced tube currents were determined using different approaches: (i) fixed mA values or ranges, or (ii) reference mA values or ranges in the case of ATCM. Reporting of mA was heterogeneous, including reference values, mean or median values, and ranges. Thus, statistics on the reported numbers would not be meaningful to present. As an alternative or in addition to tube current, some studies reported tube current-time products, which take into account the exposure time. As tube current-time product is proportional to dose, the reported mAs values can to a certain extent be considered a measure of radiation exposure.

Image reconstruction by FBP was reportedly used in 20 studies. The use of IR gradually increased with the year of the publication, including IR in image space (IRIS, *n* = 2), HIR (*n* = 23), and MBIR (*n* = 6). IR was used to create LD protocols and compared to SD protocols with FBP in 13 studies [27, 30–41]. The reconstruction technique was not reported in 10 studies. These studies were published in 2016 or earlier, making it reasonable to assume that FBP was used.

### Dose Reporting and Dose Reduction Calculation

Studies reported doses as CTDI_vol_ (*n* = 33), DLP (*n* = 29), and E (*n* = 26). Specifically, E is derived by multiplying the DLP with a conversion factor for a specific CT examination and is commonly regarded as the most appropriate indicator of stochastic radiation risk. Different DLP to E conversion factors were used from previous publications, which depended on patient age, scanned anatomical region, acquisition parameters, and time of publication. Comparable conversion factors for the head between 0.0021 and 0.0024 mSv/(mGy * cm) were used in the majority of studies [42–46], while two studies used conversion factors for the head and neck of 0.0031 and 0.0048 mSv/(mGy * cm) [38, 41], and one study used a dedicated dose calculation tool for DLP to E conversion [27].

Dose reductions were explicitly reported or retrospectively calculated from reported dose values. Achieved dose reductions ranged from 10–87%, not taking into account simulated LD studies. Two studies used simulated LD data, either by virtually lowered tube currents [47] or artificial noise insertion into CT projection data [48]. Dose values were reported as mean (with or without standard deviation) or median (with or without minimum, maximum, and interquartile ranges). The mean was extracted in favor of median when both values were provided. In Tables 1, 2, 3, 4 and 5 dose values are provided for the SD group(s), LD group(s), and other subgroups (CM volume, used CT system) where reasonably applicable.

**Table 1:** Dose reduction in NCCT of cerebral ischemia and stroke

Author	Year	Subjects (*n*)	Comparison	CT system	Acquisition parameters	Reconstruction name (level)	Dose reduction (%)	CTDI_vol_ (mGy)	DLP (mGy * cm)	E (mSv)
Paprottka [49]	2021	131 131^SD^ 131^LD^	SD vs. LD	128-MDCT (Philips Ingenuity Core, Philips Healthcare, Best, The Netherlands)	120 kV, 343 mA, 300 mAs^SD^ 120 kV, 229 mA, 200 mAs^LD^	HIR (iDose 4) MBIR (IMR 3)	33^CTDI^ 34^DLP^	46.6^SD^ 31.2^LD^	673.6^SD^ 441.9^LD^	NA
Kapur [50]	2021	423 391^SD^ 32^LD^	SD vs. LD	256-MDCT (Philips iCT, Philips Healthcare, Best, The Netherlands)	120 kV, 330 mAs^SD^ 120 kV, 280 mAs^LD^	HIR (iDose 4)	10^CTDI^ 8^DLP^ 8^E^	52.4^SD^ 47.4^LD^	1061.9^SD^ 972.9^LD^	2.6^SD^ 2.4^LD^
Bricout [51]	2015	26 13^SD^ 13^LD^	SD vs. LD	64-MDCT (Siemens SOMATOM Definition AS, Siemens Healthineers, Erlangen, Germany)	120 kV, 350 mAs^am, SD^ 100 kV, 400 mAs^am, LD^	HIR (SAFIRE 1)	23^CTDI^ 21^DLP^	42.6^SD^ 33.0^LD^	662.0^SD^ 524.0^LD^	1.4^SD^ 1.1^LD^
Bodelle [52]	2015	51 30^SD^ 21^LD^	SD^FBP^ vs. LD^FBP, HIR^	256/128-MDCT (Siemens SOMATOM Definition Flash/AS, Siemens Healthineers, Erlangen, Germany)	120 kV, 340 mAs^SD^ 120 kV, 260 mAs^LD^	FBP HIR (SAFIRE 1–5)	22^E^	NA	NA	2.3^SD^ 1.8^LD^
Ben-David [53]	2014	30 30^LD^ 30^SD^	SD vs. LD	64-MDCT (Philips Brilliance, Philips Healthcare, Best, The Netherlands)	120 kV, 445 mAs^SD^ 80 kV, 1200 mAs^LD^	NA	22^CTDI^	59.0^SD^ 46.0^LD^	NA	NA
Zacharia [54]	2011	200 100^SD^ 100^LD^	SD vs. LD	16-MDCT (Siemens Sensation, Siemens Healthineers, Erlangen, Germany)	120 kV, 300 mAs^SD^ 120 kV, 300 mAs^am, LD^	NA	36^CTDI^ 35^DLP^	NA	NA	NA

*am* automatic tube current modulation, *CTDI* based on volumetric CT dose index, *E* based on effective dose, *FBP* filtered back projection, *HIR* hybrid iterative reconstruction, *LD* low-dose, *MBIR* model-based iterative reconstruction, *SD* standard-dose

**Table 2:** Dose reduction in NCCT of intracranial hemorrhage

Author	Year	Subjects (*n*)	Comparison	CT System	Acquisition parameters	Reconstruction name (level)	Dose reduction (%)	CTDI_vol_ (mGy)	DLP (mGy * cm)	E (mSv)
Fletcher [48]	2019	83	SD vs. LD^S2^	128-MDCT (Siemens SOMATOM Definition FLASH/AS+, Siemens Healthineers, Erlangen, Germany)	120 kV, 250 eff. mAs ^SD^ 120 kV, 25–200 eff. mAs^LD^	FBP HIR (SAFIRE 2)	20–90	38.1^SD^ 3.8–30.5^LD^	NA	NA
Scholtz [55]	2017	123 36^SD, SECT^ 30^SD, DECT^ 32^LD, SECT^ 25^LD, DECT^	SD^SECT^ vs SD^DECT^ vs. LD^SECT^ vs. LD^DECT^	192-MDCT (Siemens, Siemens Healthineers, Erlangen, Germany)	120 kV, 270 mAs ^SECT^ 80/150 kV, 410/273 mAs ^DECT^ 120 kV, 270 mAs^am, SECT^ 80/150 kV, 410/273 mAs^am, DECT^	MBIR (ADMIRE)	18^CTDI, SECT^ 27^CTDI, DECT^ 25^DLP, SECT^ 24^DLP, DECT^	39.5^SD, SECT^ 41.0^SD, DECT^ 32.2^LD, SECT^ 30.0^LD, DECT^	771.5^SD, SECT^ 770.6^SD, DECT^ 575.0^LD, SECT^ 587.0^LD, DECT^	NA
Kaul [37]	2016	78 18^SD^ 22^LD1^ 20^LD2^ 18^LD3^	SD^FBP^ vs. LD1^FBP^ vs. LD2^ASIR20^ vs. LD3^ASIR30/40^	64-MDCT (GE Lightspeed VCT, GE Healthcare, Milwaukee, WI, USA)	120 kV, 100–300 mA^am, SD^ 100 kV, 100–300 mA^am, LD1–3^	FBP HIR (ASIR 20–40%)	20/43/66^CTDI^ 23/34/64^DLP^	31.9^SD^ 25.4^LD1^ 18.3^LD2^ 10.8^LD3^	396.0^SD^ 304.0^LD1^ 260.0^LD2^ 141.0^LD3^	NA
Bodelle [56]	2014	94 54^SD^ 40^LD^	SD vs. LD	128-MDCT (Siemens SOMATOM Definition Flash/AS, Siemens Healthineers, Erlangen, Germany)	120 kV, 340 mAs^SD^ 120 kV, 260 mAs^LD^	FBP HIR (SAFIRE 1–5)	29^DLP^	NA	1045.0^SD^ 744.0^LD^	2.4^SD^ 1.7^LD^

*am* automatic tube current modulation, *ASIR20/30/40* adaptive statistical iterative reconstruction at level 20/30/40% (available options: 0–50% in 10% increments), *CTDI* based on CTDI_vol_, *DECT* dual energy CT, *LD* low-dose, *LD1* low-dose protocol 1, *LD2* low-dose protocol 2, *LD3* low-dose protocol 3, *S2* 10/20/50/80% of SD corresponding to 25/50/100/200 eff. mAs using simulated noise insertion into CT projection data, *SD* standard-dose, *SECT* single energy CT

**Table 3:** Dose reduction in other NCCT of the head

Author	Year	Subjects (*n*)	Comparison	CT System	Acquisition parameters	Reconstruction name (level)	Dose reduction (%)	CTDIvol (mGy)	DLP (mGy * cm)	E (mSv)
Kaul [36]	2016	177 71^SD^ 86^LD1^ 20^LD2^	SD^FBP^ vs. LD1^ASIR20^ vs. LD2^ASIR30^	64-MDCT (GE Lightspeed VCT, GE Healthcare, Milwaukee, WI, USA)	120 kV, 100–300 mA^am, SD^ 120 kV, 100–300 mA^am, LD^	FBP HIR (ASIR 20%, 30%)	41^LD1^ 73^LD2^	51.6^SD^ 30.2^LD1^ 13.9^LD2^	768.0^SD^ 455.0^LD1^ 204.0^LD2^	1.6^SD^ 1.1^LD1^ 0.4^LD2^
Ozdoba [34]	2014	75 50^SD^ 25^LD^	SD^FBP^ vs. LD^HIR^	16/64-MDCT (Siemens SOMATOM Sensation, Siemens Healthineers, Erlangen, Germany)^SD^ 128-MDCT (Siemens Somatom Definition Edge, Siemens Healthineers, Erlangen, Germany)^LD^	120 kV, 220/380 mAs^SD^ 100 kV, 230 mAs^LD^	FBP HIR (SAFIRE)	40	57.2^SD^ 34.9^LD^	964.0^SD^ 574.1^LD^	NA
Korn [33]	2013	60 30^SD^ 30^LD^	SD^FBP^ vs. LD^FBP, HIR^	128-MDCT (Siemens Somatom Definition Flash, Siemens Healthineers, Erlangen, Germany)	120 kV, 320 mAs^SD^ 120 kV, 255 mAs^LD^	FBP HIR (SAFIRE 3)	20	59.7^SD^ 47.8^LD^	1042.0^SD^ 829.0^LD^	2.2^SD^ 1.7^LD^
Kilic [28]	2013	305 152^SD^ 153^LD^	SD^FBP^ vs. LD^ASIR^	16-MDCT (GE Lightspeed VCT)	100/120 kV, 200–420 mA^SD^ 100/120 kV, 100–290 mA^LD^	FBP HIR (ASIR 30%)	30	38.8^pf^/29.0^cer SD^ 27.7^pf^/20.2^cer LD^	466.5^SD^ 329.2^LD^	2.2^SD^ 1.5^LD^
Korn [57]	2012	90 30^SD^ 30^LD1^ 30^LD2^	SD vs. LD1 vs. LD2	128-MDCT (Siemens SOMATOM Definition Flash, Siemens Healthineers, Erlangen, Germany)	120 kV, 320 mAs^SD^ 120 kV, 275 mAs ^LD1^ 120 kV, 225 mAs ^LD2^	FBP IRIS	15^SD-LD1^ 30^SD-LD2^	60.1^SD^ 51.8^LD1^ 42.3^LD2^	1043.0^SD^ 890.0^LD1^ 733.0^LD2^	2.2^SD^ 1.8^LD1^ 1.5^LD2^
Rapalino [32]	2012	150 50^SD^ 100^LD^	SD^FBP^ vs. LD^ASIR^	64-MDCT (GE Discovery CT750HD, GE Healthcare, Milwaukee, WI, USA)	120 kV, 250 mA, 175 mAs^SD^ 120 kV, 200 mA, 140 mAs^LD^	FBP HIR (ASIR 20–100%)	26	66.5^SD^ 49.7^LD^	1270.3^SD^ 932.3^LD^	2.7^SD^ 2.0^LD^
Becker [31]	2012	150 50^SD^ 50^LD1^ 50^LD2^	SD^FBP^ vs. LD1^FBP^ vs. LD2^IRIS^	MDCT (Siemens Somatom Definition Flash)	120 kV, 320 mAs^SD^ 120 kV, 390 mAs^am, LD1, LD2^	FBP IRIS	24^CTDI^ 20^DLP^	60.0^SD^ 46.0^LD1^ 45.0^LD2^	887.0^SD^ 722.0^LD1^ 708.0^LD2^	NA
Kilic [30]	2011	149 51^SD^ 98^LD^	SD^FBP^ vs. LD^ASIR^	16-MDCT (GE Brightspeed, GE Healthcare, Milwaukee, WI, USA)	140^pf^/120^cer^ kV, 170^pf^/270^cer^ mA ^SD^ 140^pf^/120^cer^ kV, 125^pf^/190^cer^ mA ^SD^	FBP HIR (ASIR 30%)	26^pf, CTDI^ 35^cer, CTDI^ 31^DLP^	93.5^pf, SD^ 59.4^cer, SD^ 69.1^pf, LD^ 38.6^cer, LD^	1081.3^SD^ 748.6^LD^	2.3^SD^ 1.6^LD^

*am* automatic tube current modulation, *ASIR* adaptive statistical iterative reconstruction, *ASIR20/30* adaptive statistical iterative reconstruction at level 20/30/40% (available options: 0–50% in 10% increments), *cer* cerebrum, *CTDI* based on CTDI_vol_, *FBP* filtered back projection, *HIR* hybrid iterative reconstruction, *IRIS* iterative reconstruction in image space, *LD* low-dose, *LD1* low-dose protocol 1, *LD2* low-dose protocol 2, *pf* posterior fossa, *SAFIRE* sinogram-affirmed iterative reconstruction, *SAFIRE1–5* SAFIRE at level 1–5, *SD* standard-dose, *SD-LD1* dose reduction between standard-dose protocol and low-dose protocol 1, *SD-LD2* dose reduction between standard-dose protocol dose and low-dose protocol

**Table 4:** Dose reduction in CTA of intracranial aneurysm

Author	Year	Subjects (*n*)	Comparison	CT System	Acquisition parameters	Reconstruction name (level)	Dose reduction (%)	CTDI_vol_ (mGy)	DLP (mGy * cm)	E (mSv)
Chen [40]	2017	100 50^SD^ 50^LD^	SD^FBP, SC^ vs. LD^HIR, LC^	128-MDCT (Siemens SOMATOM Definition Flash, Siemens Healthineers, Erlangen, Germany)	120 kV^am, SD^ 70 kV^am, LD^	FBP HIR (SAFIRE 4)	81^CTDI^ 82^DLP^	33.4^SD^ 6.4^LD^	629.1^SD^ 116.0^LD^	1.3^SD^ 0.2^LD^
Nagayama [39]	2017	75 37^SD^ 38^LD^	SD^FBP, SC^ vs. LD^HIR, LC^	128-MDCT (Siemens SOMATOM Definition AS+, Siemens Healthineers, Erlangen, Germany)	120 kV, 350 mAs^am, SD^ 80 kV^ATVS^, 431 mAs^am, LD^	FBP HIR (SAFIRE 3)	65^CTDI^ 62^E^	41.8^SD^ 14.8^LD^	NA	1.6^SD^ 0.6^LD^
Ni [58]	2016	204 102^SD^ 102^LD^	SD^SC^ vs. LD^LC^	128-MDCT (Siemens SOMATOM Definition, Siemens Healthineers, Erlangen, Germany)	120 kV, 230 mA^am, SD^ 80 kV, 230 mA^am, LD^	NA	73^CTDI^ 73^DLP^	25.9^SD^ 7.0^LD^	507.0^SD^ 136.7^LD^	1.1^SD^ 0.3^LD^
Yang [59]	2016	80 40^SD^ 40^LD^	SD vs. LD	128-DS-MDCT (Siemens SOMATOM Definition Flash)	80/sn140 kV, 300/150 mAs^SD^ 80/sn140 kV, 200/100 mAs^LD^	FBP HIR (SAFIRE 3)	34^CTDI^ 31^DLP^	20.6^SD^ 13.6^LD^	378.3^SD^ 259.4^LD^	0.8^SD^ 0.5^LD^
Tang [60]	2015	294 148^SD^ 146^LD^	SD vs. LD	64-MDCT (GE Lightspeed, GE Healthcare, Milwaukee, WI, USA)	120 kV, 641 mA^SD^ 100 kV, 380 mA^LD^	NA	36^CTDI^ 36^DLP^	55.7^SD^ 35.9^LD^	932.6^SD^ 594.8^LD^	2.0^SD^ 1.3^LD^
Chen [35]	2015	100 50^SD^ 50^LD^	SD^FBP^ vs. LD^HIR^	128-MDCT (Siemens SOMATOM Definition Flash)	120 kV^am, SD^ 70 kV^am, LD^	FBP HIR (SAFIRE 1–5)	80^CTDI^ 81^DLP^	33.7^SD^ 6.6^LD^	609.9^SD^ 118.0^LD^	1.3^SD^ 0.2^LD^
Luo [61]	2014	120 40^SD^ 40^LD1^ 40^LD2^	SD vs. LD1 vs. LD2	128-MDCT (Siemens SOMATOM Definition)	120 kV, 230 mAs^am^, 70 ml CM ^SD^ 100 kV, 230 mAs^am^, 30 ml CM ^LD1^ 80 kV, 230 mAs^am^, 30 ml CM ^LD2^	NA	45^SD-LD1, CTDI^ 73^SD-LD2, CTDI^ 51^LD1-LD2, CTDI^ 44^SD-LD1, DLP^ 74^SD-LD2, DLP^ 52^LD1-LD2, DLP^	26.1^SD^ 14.4^LD1^ 7.0^LD1^	515.0^SD^ 286.0^LD^ 136.0^LD2^	1.1^SD^ 0.6^LD^ 0.3^LD2^
Kidoh [62]	2013	98 32 ^SD, SC^ 33 ^SD, LC^ 33 ^LD, LC^	SD vs. LD SC vs. LC	256-MDCT (Brilliance iCT, Philips Healthcare, Best, The Netherlands)	100 kV, 641 mA, 800 eff. mAs ^SD^ 80 kV, 923 mA, 1365 eff. mAs^LD^ 370 mg/kg CM ^SC^ 296 mg/kg CM ^LC^	NA	16^CTDI^	59.2^SD^ 49.7^LD^	NA	NA
Sun [63]	2012	48 24^SD^ 24^LD^	SD vs. LD	320-MDCT (Aquilion One, Toshiba Medical Systems, Canon Medical Systems Corporation, Ōtawara, Tochigi, Japan)	120 kV, 200 mA, 150 mAs^SD^ 80 kV, 200 mA, 150 mAs^LD^	NA	70^CTDI^ 70^DLP^	54.6^SD^ 16.6^LD^	876.6^SD^ 265.6^LD^	1.8^SD^ 0.6^LD^
Waaijer [64]	2007	40 20^SD^ 20^LD^	SD vs. LD	16 MDCT (MX 8000 IDT, Philips Healthcare, Best, The Netherlands)	120 kV, 200 mAs^SD^ 90 kV, 330 mAs^LD^	NA	24	27.2^SD^ 20.6^LD^	NA	NA

*am* automatic tube current modulation, *ATVS* Automated tube voltage selection with 120 kV reference, *CM* contrast medium, *CTDI* based on CTDI_vol_, *E* based on effective dose, *FBP* filtered back projection, *HIR* hybrid iterative reconstruction, *LC* low contrast medium concentration, *LD* low-dose, *LD1* low-dose protocol 1 using 120 kV and 150 mAs, *LD2* low-dose protocol 2 using 100 kV and 230 mAs, *RD* reference dose using 120 kV, *RD-SD* dose reduction between reference-dose protocol and standard-dose protocol, *RD-LD* dose reduction between reference-dose protocol and low-dose protocol, *SAFIRE* sinogram-affirmed iterative reconstruction, *SAFIRE1–5* SAFIRE at level 1–5, *SC* standard contrast medium concentration, *SD* standard-dose, *SD-LD* dose reduction between standard-dose protocol and low-dose protocol, *SD-LD1* dose reduction between standard-dose protocol and low-dose protocol 1, *SD-LD2* dose reduction between standard-dose protocol dose and low-dose protocol 2, *sn140* use of a selective proton shield (SPS) with a 140 kV X-ray tube with additional tin filtration, *LD1-LD2* dose reduction between low-dose protocol 1 and low-dose protocol 2

**Table 5:** Dose reduction in CTA of other cerebrovascular diseases

Author	Year	Subjects (*n*)	Comparison	CT System	Acquisition parameters	Reconstruction name (level)	Dose reduction (%)	CTDI_vol_ (mGy)	DLP (mGy * cm)	E (mSv)
Sollmann [47]	2019	30	SD vs. LD^S1^	128-MDCT (Philips Ingenuity Core; Philips Healthcare, Best, The Netherlands)	120 kV, 263 mA, 130 mAs^am,^ ^SD^ 13–65 mAs^LD^	HIR (SIR)	50–90	8.5^SD^ 0.9–4.2^LD^	NA	NA
Annoni [65]	2019	205 100^SD^ 105^LD^	SD vs. LD	64-MDCT (GE Discovery 750 HD, GE Healthcare, Milwaukee, WI, USA)^SD^ 256-MDCT (GE Revolution, GE Healthcare, Milwaukee, WI, USA)^LD^	100 kV, 213–600 mA^am, SD^ 80 kV, 120–500 mA^am, LD^	HIR (ASIR 50%) ^SD^ HIR (ASIR‑V 50%)^LD^	87^CTDI^ 86^DLP^	20.5^SD^ 2.7^LD^	735.3^SD^ 100.7^LD^	1.6^SD^ 0.2^LD^
Wang [66]	2018	101 53^SD^ 48^LD^	SD vs. LD	256-MDCT (Philips Brilliance iCT; Philips Healthcare, Best, The Netherlands)	100 kV, 220 mAs^SD^ 80 kV, 220 mAs^LD^	FBP HIR (iDose 4) MBIR (IMR 1)	51^SD-LD^ 39^RD-SD^ 70^RD-LD^	16.9^SD^ 8.3^LD^ 27.6^RD^	540.0^SD^ 267.2^LD^ 884.4^RD^	1.1^SD^ 0.6^LD^ 1.9^RD^
Chen [41]	2018	121 39^SD^ 41^LD1^ 41^LD2^	SD^FBP^ vs. LD1^ASIR‑V^ vs. LD2 ^ASIR‑V^	256-MDCT (GE Revolution)	120 kV, 10–350 mA^am, SD^ 120 kV, 10–350 mA^am, LD1^ 100 kV, 10–400 mA^am, LD2^	FBP HIR (ASIR‑V 50%)	24^SD-LD1, CTDI^ 37^SD-LD2, CTDI^ 25^SD-LD1, DLP^ 40^SD-LD2, DLP^	9.1^SD^ 6.9^LD1^ 5.7^LD2^	393.0^SD^ 293.0^LD1^ 236.0^LD2^	9.1^SD^ 6.9^LD1^ 5.7^LD2^
Leithner [67]	2018	43	SD vs. LD	192-MDCT (Siemens SOMATOM Force, Siemens Healthineers, Erlangen, Germany)	120 kV, 95/59 mAs^am, SD^ 90 kV, 95 mAs^am, LD^	MBIR (ADMIRE 3)	40^DLP^	2.8^SD^ 1.7^LD^	185.4^SD^ 110.6^LD^	0.4^SD^ 0.2^LD^
Chen [38]	2017	50 25^SD^ 25^LD^	SD^SC, FBP^ vs. LD^LC, MBIR^	192-MDCT (Siemens SOMATOM Force)	100 kV, 700 mAs^am^, 40 ml/kg CM ^SD^ 70 kV, 700 mAs^am^, 25 ml CM^LD^	FBP MBIR (ADMIRE 3)	54^CTDI^ 56^DLP^	4.6^SD^ 2.1^LD^	190.8^SD^ 84.2^LD^	0.9^SD^ 0.4^LD^
Kayan [68]	2016	101 50^SD^ 51^LD^	SD^SC^ vs. LD^LC^	192-MDCT (Siemens Definition AS, Siemens Healthineers, Erlangen, Germany)	100 kV, 150 mA^am^, 1 ml/kg CM ^SD^ 80 kV, 150 mA^am^, 0.5 ml/kg CM^LD^	NA	48^DLP^	NA	225.7^SD^ 116.6^LD^	NA
Moloney [27]	2016	20	SD^FBP, ASIR^ vs. LD^ASIR^ vs. LD^MBIR^	64-MDCT (GE Lightspeed VCT, GE Healthcare, Milwaukee, WI, USA)	100 kV, 60–230 mA^am, SD^ 100 kV, 30–150 mA^am, LD^	FBP HIR (ASIR) MBIR (Veo)	50	NA	688.0^SD^ 341.3^LD^	1.8^SD^ 3.7^LD^
Bricout [51]	2015	26 13^SD^ 13^LD^	SD^SAFIRE1^ vs. LD^SAFIRE3^	64-MDCT (Siemens SOMATOM Definition AS, Siemens Healthineers, Erlangen, Germany)	100 kV, 250 mAs^am, SD^ 100 kV, 220 mAs^am, LD^	HIR (SAFIRE 1, 3)	31^CTDI^ 26^DLP^	24.5^SD^ 17.0^LD^	369.0^SD^ 273.0^LD^	0.8^SD^ 0.6^LD^
Bahner [29]	2005	29	SD vs. LD	SDCT (Siemens SOMATOM Plus 4, Siemens Healthineers, Erlangen, Germany)	120 kV, 200 mA, 150 mAs^SD^ 80 kV, 255 mA, 255 mAS^LD^	NA	38^CTDI^ 43^E^	21.9^SD^ 13.5^LD^	NA	0.7^SD^ 0.4^LD^

*ADMIRE* advanced modeled iterative reconstruction, *am* automatic tube current modulation, *ASIR* first generation version of ASIR, *ASIR‑V* third generation version of ASIR, *CM* contrast medium, *CTDI* based on CTDI_vol,_ *E* based on effective dose, FBP filtered back projection, *HIR* hybrid iterative reconstruction, *LC* low contrast medium concentration, *LD* low-dose, *LD1* low-dose protocol 1, *LD2* low-dose protocol 2, *MBIR* model-based iterative reconstruction, *RD* reference dose protocol, *S1* 10/25/50% of SD using simulated lower tube currents, *SC* standard contrast medium concentration, *SD* standard-dose, *SD-LD* dose reduction between standard-dose protocol and low-dose protocol, *SD-LD1* dose reduction between standard-dose protocol and low-dose protocol 1, *SD-LD2* dose reduction between standard-protocol dose and low-dose protocol 2, *SIR* statistical iterative reconstruction

### Outcome Measures

#### Quantitative Measures

Quantitative outcome measures included physical metrics of objective image noise and contrast as well as other quantitative parameters. In total, 34 studies reported on quantitative image noise as the standard deviation of Hounsfield units (*n* = 27) and/or signal-to-noise ratio (SNR, *n* = 29), measured in one or multiple standardized regions of interest (ROIs). For NCCT, most frequent ROI locations were cortical and nuclear gray matter, white matter, and cerebrospinal fluid (CSF). For CTA, most frequent ROI locations were the lumen of large intracranial arteries (anterior cerebral artery, ACA; middle cerebral artery, MCA; posterior cerebral artery, PCA; basilar artery, BA), internal carotid arteries (ICA), and common carotid arteries (CCA), as well as adjacent muscles and brain parenchyma. Contrast-to-noise ratio (CNR) was reported in 29 studies and was usually determined between gray and white matter for NCCT, and between arterial lumen and adjacent brain parenchyma or muscles for CTA. Other quantitative parameters were reported in 8 CTA studies, including arterial attenuation measured in HU (*n* = 4, [29, 64, 66, 68]) and aneurysm diameters (*n* = 4, [58–60, 63]).

#### Qualitative Measures

Purely quantitative outcome measures are important to enable a comparable IQ assessment [69]. However, more subjective outcome measures are needed to assess the utility of NCCT and CTA at different doses for the clinical application or diagnostic question. The most frequently reported qualitative measures comparable across all included studies were subjective IQ (*n* = 26), containing common subcategories for some studies (e.g., overall IQ, artifacts, image contrast and sharpness), followed by subjective image noise (*n* = 16) and diagnostic confidence (*n* = 15, alternatively termed diagnostic utility, ability, reliability, or acceptability). These measures usually used 3–5 point Likert scales. Furthermore, other application-specific variables were evaluated as qualitative outcome measures and are described in the corresponding sections. In 24 studies, qualitative items were rated by 2 or more readers, and inter-observer agreement (IOA) was reported using the intraclass correlation coefficient (ICC), Cohen’s kappa, or Fleiss’ kappa [70, 71]. In the majority of studies IOA was at least substantial (> 0.6).

For NCCT, two studies assessed diagnostic performance for findings corresponding to acute neurologic deficits [48] or for the detection of ICH [55], reporting classification metrics (accuracy, sensitivity, and specificity) as well as reader agreement rules and jackknife alternative free-response receiver operating characteristic figure of merit (JAFROC-FOM) [48, 72].

For CTA, diagnostic performance for the detection and size measurements of intracranial aneurysms was assessed in 7 studies, using digital subtraction angiography (DSA) as the reference standard. Reported metrics included classification metrics (*n* = 4; [58, 60, 61, 63]), aneurysm detection numbers or rates (*n* = 5; [35, 40, 59, 60, 63]), and correlation coefficients of aneurysm diameters (*n* = 3; [59, 60, 63]), 5 studies [27, 38, 65, 67, 68] investigated the grade of stenosis of the ICA or extracranial and intracranial arteries using the European Carotid Surgery Trial (ECST) [73] or North American Symptomatic Carotid Endarterectomy Trial (NASCET) [74] criteria. Stenosis gradings were used for subgroup analyses and assessment of diagnostic performance.

### Dose Reduction in NCCT

Dose reduction in NCCT was considered in 18 articles, including 6 studies focusing on ischemia and stroke and 4 studies focusing on ICH. The remaining 8 NCCT studies had unspecified or mixed indications, e.g., evaluation of trauma, skull fracture, amnesia, loss of consciousness, seizure, headache, vomiting, focal neurological deficit, coagulopathy, treatment with anticoagulants, or tumor staging. For two studies comparing SD and LD protocols of multi-modal CT (NCCT, CTA, and CTP), only the NCCT results were extracted [50, 51]. The results are summarized in Tables 1, 2 and 3.

#### Cerebral Ischemia and Stroke

Dose reductions ranged from 10 to 36% without impairment of IQ (based on 4 studies, [50–52, 54]). Reported doses in terms of CTDI_vol_ and DLP ranged from 31.2 to 59.0 mGy and 441.9–1061.9 mGy * cm, respectively. The highest dose reduction of 36% was reported in 200 patients by Zacharia et al., who compared SD and LD protocols by using ATCM in z‑direction for the LD protocol [54].

**Fig. 2:**
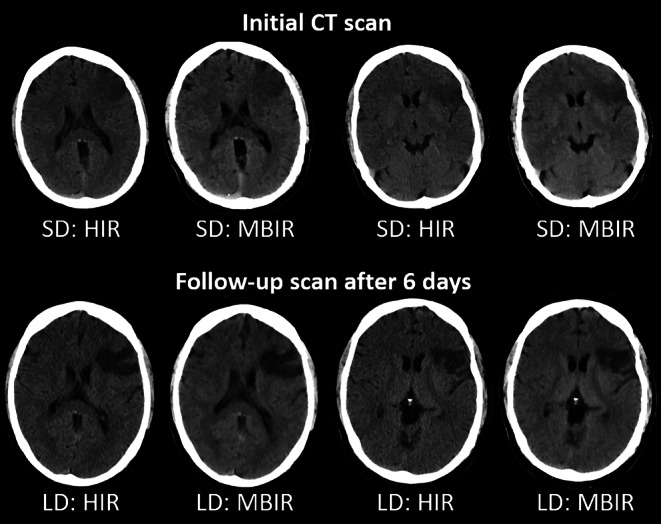
Initial SD NCCT (*upper row*) and follow-up LD NCCT (*lower row*) of a 41-year-old man with left-sided MCA infarction. Axial images were reconstructed using HIR and MBIR. The demarcated area in the left hemisphere shows better conspicuousness due to higher CNR with MBIR compared to HIR. *CNR* contrast-to-noise ratio, *HIR* hybrid iterative reconstruction, *LD* low-dose, *MBIR* model-based iterative reconstruction, *MCA* middle cerebral artery, *NCCT* non-contrast computed tomography, *SD* standard-dose

In the context of stroke imaging, the detection of ischemic lesions is an important indication of NCCT. In comparison to SD-CT, the conspicuousness of ischemic demarcation could be shown to be preserved by using HIR instead of FBP [52], or it could even be improved by using MBIR instead of HIR for LD-CT (Fig. 2; [49]). Furthermore, excellent IOA (range: 0.80–0.93) regarding overall IQ, anatomic detail, gray-white matter differentiation, and conspicuousness of ischemic demarcation was demonstrated at reduced dose [49, 50, 52]. Furthermore, gray to white matter contrast per dose was found to be markedly increased in the context of acute stroke scans using a reduced dose protocol [53].

#### Intracranial Hemorrhage

Four NCCT studies focused on or included patients for ICH evaluation, using heterogeneous study designs and methodologies. Not taking into account simulated LD protocols, dose reductions ranged from 18 to 66% while maintaining sufficient quantitative and qualitative IQ [37, 48, 55, 56].

Reported doses in terms of CTDI_vol_ and DLP ranged from 10.8–41.0 mGy and 141.0–1045.0 mGy * cm, respectively. The highest dose reduction of 66% was reported by Kaul et al., who compared an SD and multiple LD protocols by using reduced tube voltage and ATCM combined with increasing levels of HIR in pediatric patients. A dose reduction of 34% still provided adequate image quality and diagnosis-related confidence. The corresponding LD protocol was therefore recommended for everyday clinical practice [37].

In a study of 94 consecutive ICH patients by Bodelle et al., HIR was recommended over FBP for the evaluation of brain structures and ICH detection when using an LD protocol, enabling a considerable dose reduction of 29% [56].

Scholtz et al. compared SD to LD protocols using a dual-source MDCT in single-energy (SECT) and dual-energy (DECT) mode. With respect to ICH detection, both SECT and DECT achieved excellent sensitivity and specificity at a significant dose reduction of about 25%. The authors recommended the routine use of ATCM and MBIR to reduce dose in ICH evaluation for both SECT and DECT [55].

By inserting artificial noise into MDCT projection data acquired at 250 effective mAs, Fletcher et al. generated LD images corresponding to 25–200 effective mAs using FBP and HIR. Diagnostic performance assessed by JAFROC-FOM was shown to be non-inferior for the 100 effective mAs LD data reconstructed with HIR. As nowadays IR can be considered the standard reconstruction method for most centers, a dose reduction potential of 60% can be derived from this study [48].

#### NCCT Without Specified Indications

The remaining NCCT studies included patients with mixed or unspecified indications. All 8 studies reported maintained or even improved subjective IQ that was diagnostically acceptable while achieving dose reductions ranging mainly from 20 to 40% [28, 30–34, 57]. In all studies, this was achieved with (mainly hybrid) IR. The commonly used objective measures of IQ were SNR and CNR, primarily assessed within or between gray and white matter, and additionally within the CSF space in a subset of studies [28, 30, 31, 33, 57]. Comparing LD and SD protocols, SNR was slightly decreased to unchanged, while CNR showed mostly no relevant differences. Reported doses in terms of CTDI_vol_ and DLP ranged from 13.9–93.5 mGy and 204.0–1270.3 mGy * cm, respectively. The highest dose reduction was 73% using a strong level of HIR (30% with ASIR; GE Healthcare, Milwaukee, WI, USA) in the LD protocol compared to FBP in the SD protocol [36]. Furthermore, reported IOA ranged from moderate (0.54–0.60) regarding noise, sharpness, and diagnostic acceptability [30], to substantial (0.68–0.71) regarding overall IQ [33, 57], artifacts [30], and diagnosis-related confidence [36], to excellent (0.85–0.6) regarding noise, contrast and overall diagnosability [36]. Non-standard IQ evaluation parameters used without dedicated reported IOA included distinctness of posterior fossa contents [57] and visibility of small structures [32].

### Dose Reduction in CTA of the Head and Neck

Dose reduction in CTA of the head and neck was considered in 20 articles, 10 studies investigated intracranial aneurysms, including 5 studies that used a lower CM volume in the LD group [39, 40, 58, 61, 62]. Another 10 studies focused on other cerebrovascular diseases, including 2 studies that used a lower CM volume in the LD group [38, 68]. For one study comparing SD and LD protocols of multi-modal CT (NCCT, CTA, and CTP), only the CTA results were extracted [51]. Results are summarized in Tables 4 and 5.

#### Intracranial Aneurysms

Dose reductions ranged from 16–82%, and none of the studies showed a relevant impairment of subjective IQ for the LD compared to the SD protocol. Specifically, studies using lower CM volume in the LD group consistently demonstrated improved IQ [39, 40, 58, 61, 62]. Reported doses in terms of CTDI_vol_ and DLP ranged from 6.4–59.2 mGy and 116.0–932.6 mGy * cm, respectively. The highest dose reduction of 82% was reported by Chen et al., who used HIR for the LD protocol acquired with reduced tube voltage and CM volume [40].

In addition to the detection of aneurysms of the brain-supplying arteries, aneurysm size measurements provide relevant information derived from CTA. Diameters were shown to be unaffected in LD-CTA compared to SD-CTA [59], and not significantly different from DSA measurements for both LD-CTA and SD-CTA [63]. DSA is the reference standard for the evaluation of aneurysms and was used in four studies to assess diagnostic performance. High diagnostic accuracy (90–99% for LD-CTA vs. 94–100% for SD-CTA), sensitivity (80–95% vs. 91–100%), and specificity (93–100% vs. 93–100%) without statistically significant differences between each CTA protocol and DSA, or between the two CTA protocols were demonstrated [58, 60, 61, 63]. In a different approach to assess diagnostic capabilities, Yang et al. used surgical verification as reference, and 29/29 (100%) of the aneurysms in the LD group were detected compared to 25/26 (96%) in the SD group [59].

Furthermore, IOA regarding overall IQ and other qualitative outcome measures was substantial to excellent (0.60–1.00) in the majority of studies. Other subjective outcome measures included vascular sharpness [35, 39, 40], noise [40], arterial contrast [39], calcifications [35] as well as visibility of blood vessels (in particular small arteries, arteries near the skull base and surgical clips, and peripheral veins) [39, 62].

#### Other Cerebrovascular Diseases

Not taking into account simulated LD protocols, dose reductions ranged from 25 to 87% [27, 38, 41, 51, 65–68], with the majority of studies reporting maximum values of 40–70% [27, 38, 41, 66–68] while showing maintained or even improved subjective IQ that was diagnostically acceptable. Reported doses in terms of CTDI_vol_ and DLP were 1.7–24.5 mGy and 84.2–735.3 mGy * cm, respectively. The highest dose reduction of 87% was reported by Annoni et al., who used a newer version of HIR for the LD protocol acquired at reduced tube voltage [65].

Diagnostic performance with respect to ICA stenosis or extracranial and intracranial arterial stenosis was analyzed using different approaches. Two studies used DSA as the reference and reported high diagnostic accuracy (94–99%), sensitivity (91–100%), and specificity (94–99%) for LD-CTA [38, 65]. Annoni et al. additionally demonstrated an excellent correlation regarding the stenosis degree between LD-CTA and DSA (r = 0.98) [65]. Two studies showed excellent IOA (0.81–1.00) for detection and grading of ICA stenosis without a statistically significant difference between SD-CTA and LD-CTA [27, 67].

**Fig. 3:**
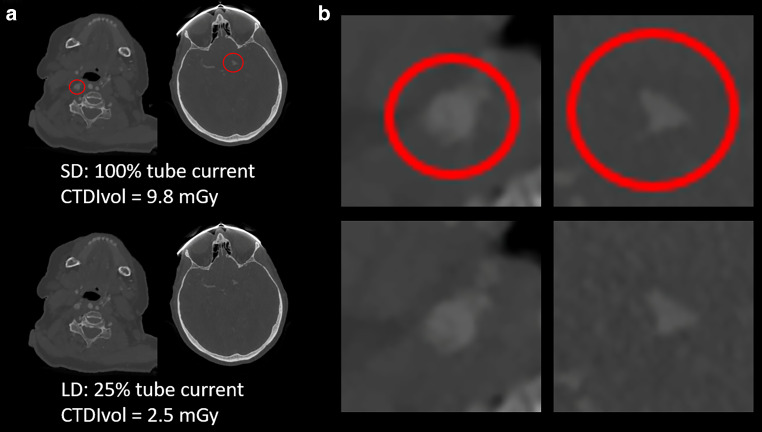
SD (100% tube current, *upper row*) and simulated LD CTA using virtual tube current reduction (25% of original tube current, *bottom row*). **a** *Left column*: 81-year-old woman with right-sided dissection of the extracranial ICA (*red circle*). *Right column*: 84-year-old man with left-sided thrombotic MCA occlusion of the M1 segment (*red circle*). **b** Magnified views of the relevant pathologies. The shown axial images were reconstructed with statistical iterative reconstruction, a type of HIR, with low regularization to generate images close to clinical appearance. *CTA* computed tomography angiography, *HIR* hybrid iterative reconstruction, *ICA* internal carotid artery, *LD* low-dose, *MCA* middle cerebral artery, *SD* standard-dose

In a different study design, Sollmann et al. applied virtually lowered tube current in 30 patients who underwent CTA. Even at 25% of the original dose, good vascular contrast and clearly detectable arteries were demonstrated (Fig. 3). As a result, with the adequate type of HIR, all large vessel occlusions or dissections (15/15) could be detected with medium to high diagnostic confidence and excellent IOA (1.00) [47]. Furthermore, IOA regarding overall IQ and diagnostic confidence was substantial to excellent (0.75–1.00) when reported in other studies on that matter [41, 65, 66].

## Discussion

In this article, dose reduction techniques for NCCT and CTA of the head were systematically reviewed and 37 studies representing the most common clinical indications were included. LD-CT and SD-CT were most frequently compared between a different combination of tube settings and image reconstruction techniques.

**Fig. 4:**
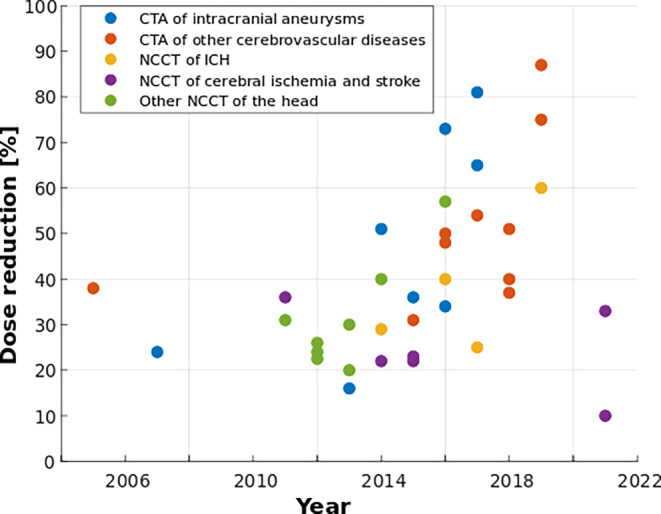
Development of dose reduction over the last two decades in computed tomography of the head. For studies reporting multiple dose reduction values or ranges, the highest value with acceptable image quality and/or diagnostic performance is displayed

For NCCT of cerebral ischemia and stroke, achieved dose reductions ranged from 10 to 36%. The majority of studies reduced the dose by at least 22% and maintained objective and subjective IQ, while the use of more advanced IR improved visualization of ischemic demarcation. For NCCT of intracranial hemorrhage, slightly higher dose reductions of 18–66% with sufficient IQ were achieved, while simulated LD-CT suggested a dose reduction potential of 60%. Achieved dose reductions for NCCT with unspecified indication were in a comparable range of 20–40% (Fig. 4).

In the context of NCCT, it has to be mentioned that the required dose for differentiation of normal from pathological tissue depends on the contrast difference. For the detection of cerebral ischemia, the contrast differences are very small and therefore a higher dose is required compared to the assessment of ICH, where the contrast difference is significantly higher.

Given the only slightly decreased or even equivalent IQ and diagnostic acceptability of LD protocols and ongoing improvements in image reconstruction, there could be more potential for future dose reduction beyond the reported 20–40% in NCCT of the head. However, increased dose reductions must be used with care. In the abdominal region, the diagnostic performance of low contrast lesions has been reported to be impaired when dose reduction exceeded 25% [75, 76]. Such low contrast lesions are key diagnostic questions in NCCT, particularly in cerebral ischemia and stroke. Diagnostic performance must therefore be thoroughly evaluated before higher dose reductions, facilitated by advanced IR or other novel reconstruction techniques, are implemented.

For CTA of intracranial aneurysms, dose reduction ranged from 16 to 82%, while 6 of 10 studies even achieved maximum values of 65% or higher (Fig. 4). Despite the substantial dose reduction, subjective IQ and diagnostic performance for aneurysm detection were non-inferior. Hence, CTA could be a less invasive and dose-intensive alternative to DSA for accurate evaluation of intracranial aneurysms. Achieved dose reduction in CTA of other cerebrovascular diseases and carotid artery disease was at a comparable level. Similarly, subjective IQ and diagnostic performance with respect to arterial stenosis were not negatively affected. Overall, it can be concluded that tube voltage reduction, advanced IR, and CM volume optimization may represent the most effective combination for dose reduction in head CTA. Beyond radiation exposure, patient care can be improved via lower contrast media-related morbidity, as long as long as CM volume is reduced with care, in order to not impair the visibility of intracranial vessels and associated diagnostic performance.

When comparing dose reductions of NCCT and CTA, one important difference regarding the required contrast has to be acknowledged: in comparison to NCCT of cerebral ischemia or ICH, blood vessels in CTA are high-contrast objects which therefore require a significantly lower dose per se.

Until now, dose reduction in head CT has mainly been achieved through modified tube settings while advanced reconstruction techniques ensured acceptable IQ and high diagnostic performance. Most of these advances have been enabled by software innovations, but current and future developments in CT hardware will also very likely increase dose reduction. The clinical introduction of photon-counting CT (PCCT) represents a revolution in CT imaging, also with respect to radiation exposure [77, 78]. At the same radiation dose as conventional CT, better gray to white matter differentiation due to higher CNR and less image noise has been demonstrated. The ensuing high potential of PCCT for IQ improvement in NCCT of the head could translate into a radiation dose reduction of approximately 40% [79]. At a comparable dose as conventional single-energy CT, PCCT has been reported to reduce beam-hardening artifacts and improve IQ in arterial segments close to surrounding bone [80]. In combination with its ability of spectral material decomposition, it thus bears great potential to further reduce radiation dose for intracranial and carotid CTA, particularly in the presence of calcifications and plaques. Furthermore, the increasing clinical use of PCCT implies the routine acquisition of spectral data. With this evolution, virtual non-contrast (VNC) scans could make NCCT scans redundant in stroke or other head CT protocols requiring non-contrast and contrast-enhanced images, and thereby save a considerable amount of radiation dose. Clinical translation of AI in CT imaging is steadily increasing, which can be expected to unveil additional dose reduction opportunities for head CT, primarily on the image reconstruction side [25–29]. Beyond the application of CNNs to accelerate MBIR algorithms, deep-learning techniques can enable the implementation of more complex functions in IR models [16]. Given that existing study results can be validated and reliably translated into the clinical routine, the use of AI will further reduce the required dose in head CT.

The present review article is not without limitations. Methodology and design of the included studies are quite heterogeneous, mostly due to the development of CT hardware and software over time, different CT manufacturers and models, and the fact that different departments have established different head CT protocols. As a result, different dose parameters (CTDI_vol_, DLP, E) with different ranges of values have been used to determine the amount of dose reduction. Furthermore, absolute definitions of SD and LD protocol cannot be reasonably established in the context of the included studies. Consequently, the start parameters for dose reduction quantification could not be normalized which potentially compromises objectivity and comparability of dose reduction.

In conclusion, considerable dose reduction in NCCT and CTA of the head can be realized by global approaches, such as decreased tube voltage and ATCM, while advances in IR algorithms ensure diagnostic image quality. For CTA examinations, the combination with lower CM volume can be particularly effective for improved patient care. In the upcoming years, the ongoing clinical transition of novel CT acquisition and reconstruction techniques can be expected to enable additional dose reductions of 40% or more, and potentially further CM volume reduction. However, it will be essential that the image quality and diagnostic performance is thoroughly evaluated to guarantee that the benefits of head CT are not compromised.

## Appendix

### PubMed Search Terms

#### Dose Reduction in NCCT of Cerebral Ischemia and Stroke:

((computed tomography) OR (CT)) AND ((low-dose) OR (low dose) OR (dose reduction) OR (low-kilovolt) OR (low kilovolt) OR (low-kV) OR (low kV) OR (iterative reconstruction)) AND ((stroke) OR (cerebral ischemia) OR (cerebral infarction) OR (cerebral infarct) OR (brain ischemia) OR (brain infarction) OR (brain infarct) OR (demarcation)).

#### Dose Reduction in NCCT of Intracranial Hemorrhage (ICH):

((computed tomography) OR (CT)) AND ((low-dose) OR (low dose) OR (dose reduction) OR (low-kilovolt) OR (low kilovolt) OR (low-kV) OR (low kV) OR (iterative reconstruction)) AND ((cerebral bleeding) OR (cerebral hemorrhage) OR (cerebral haemorrhage) OR (intracerebral hemorrhage) OR (intracerebral haemorrhage) OR (intracranial hemorrhage) OR (intracranial haemorrhage) OR (ICH) OR (brain bleeding) OR (brain hemorrhage) OR (brain haemorrhage) OR (subarachnoid bleeding) OR (subdural hematoma) OR (subdural haematoma) OR (subdural hemorrhage) OR (subdural haemorrhage) OR (SDH) OR (subdural bleeding) OR (epidural hematoma) OR (epidural haematoma) OR (epidural hemorrhage) OR (epidural haemorrhage) OR (extradural hematoma) OR (extradural haematoma) OR (epidural bleeding)).

#### Dose Reduction in CTA of Intracranial Aneurysms:

((computed tomography angiography) OR (CT angiography) OR (CTA) OR (computed tomography angiogram) OR (CT angiogram)) AND ((low-dose) OR (low dose) OR (dose reduction) OR (low-kilovolt) OR (low kilovolt) OR (low-kV) OR (low kV) OR (iterative reconstruction)) AND ((cerebral aneurysm) OR (brain aneurysm)).

#### Dose Reduction in CTA of Other Cerebrovascular Diseases and Carotid Artery Disease:

((computed tomography angiography) OR (CT angiography) OR (CTA) OR (computed tomography angiogram) OR (CT angiogram)) AND ((low-dose) OR (low dose) OR (dose reduction) OR (low-kilovolt) OR (low kilovolt) OR (low-kV) OR (low kV) OR (iterative reconstruction)) AND ((stroke) OR (cerebral ischemia) OR (cerebral infarction) OR (cerebral infarct) OR (brain ischemia) OR (brain infarction) OR (brain infarct) OR (vessel occlusion) OR (LVO) OR (thrombus) OR (vessel stenosis) OR (vessel dissection) OR (arterial stenosis) OR (arterial dissection) OR (carotid stenosis) OR (carotid dissection) OR (cerebral vascular malformation) OR (brain vascular malformation) OR (cerebral arteriovenous malformation) OR (brain arteriovenous malformation) OR (cerebral AVM) OR (brain AVM)).

## Abbreviations

ACA: Anterior cerebral artery
ADMIRE: Advanced modeled iterative reconstruction
AEC: Automatic exposure control
AI: Artificial intelligence
ALARA: As low as reasonably achievable
ASIR: Adaptive statistical iterative reconstruction
ATCM: Automatic tube current modulation
BA: Basilar artery
CCA: Common carotid artery
CM: Contrast medium
CNN: Convolutional neural network
CNR: Contrast-to-noise ratio
CT: Computed tomography
CTA: Computed tomography angiography
CTDI_vol_: Volumetric CT dose index
CTP: Computed tomography perfusion
DECT: Dual-energy CT
DLP: Dose length product
DSA: Digital subtraction angiography
E: Effective dose
ECST: European Carotid Surgery Trial
FBP: Filtered back projection
HIR: Hybrid iterative reconstruction
ICA: Internal carotid artery
ICC: Intraclass correlation coefficient
ICH: Intracranial hemorrhage
IMR: Iterative model reconstruction
IOA: Interobserver agreement
IQ: Image quality
IR: Iterative reconstruction
JAFROC-FOM: Jackknife alternative free-response receiver operating characteristic figure of merit
LD: Low-dose
LD-CT: Low-dose computed tomography
LD-CTA: Low-dose computed tomography angiography
MBIR: Model-based iterative reconstruction
MCA: Middle cerebral artery
MDCT: Multi-detector computed tomography
NA: Not available
NASCET: North American Symptomatic Carotid Endarterectomy Trial
NCCT: Non-contrast computed tomography
PCA: Posterior cerebral artery
PCCT: Photon counting CT
PRISMA: Preferred Reporting Items for Systematic reviews and Meta-Analyses
ROI: Region of interest
SAFIRE: Sinogram-affirmed iterative reconstruction
SD: Standard-dose
SD-CT: Standard-dose CT
SECT: Single-energy CT
SIR: Statistical iterative reconstruction
SNR: Signal-to-noise ratio
VNC: Virtual non-contrast
